# Effect of Heat Supplied to the Joint in the MAG Welding Process of Ferritic–Austenitic Stainless Steel 1.4462 on the Size of the Cross-Sectional Area of the Joints

**DOI:** 10.3390/ma17246192

**Published:** 2024-12-18

**Authors:** Stanisław Pałubicki, Jarosław Plichta, Krzysztof Nadolny

**Affiliations:** Department of Production Engineering, Faculty of Mechanical and Energy Engineering, Koszalin University of Technology, Raclawicka 15–17, 75-620 Koszalin, Poland; stanislaw.palubicki@tu.koszalin.pl (S.P.); jaroslaw.plichta@tu.koszalin.pl (J.P.)

**Keywords:** ferritic–austenitic stainless steel, heat input, welding discrepancies, welded joint macrostructure

## Abstract

In this study, the relationships between the values of the parameters included in heat input (welding current, arc voltage and welding speed) and their effects on the size of the cross-sectional areas of welds in joints made of ferritic–austenitic stainless steel using the GMAW method were determined. An attempt was also made to determine to what extent it will be possible to predict the properties of fabricated welded joints using the functional relationship describing the effect of the value of heat input on the size of the cross-sectional area of welds. The analysis of the developed mathematical models shows their suitability for explaining (and predicting) the sizes of the cross-sectional areas of welded joints depending on the values of the input parameters of the welding process. Determining the regression function and making a three-dimensional plot of it (response surface) can provide a starting point for optimizing the parameters of the welding process. The results have practical relevance, supporting weld quality control and process design in industrial conditions, especially in applications requiring high strength and corrosion resistance, in industries such as construction and offshore.

## 1. Introduction

Recent years have seen tremendous progress in new technological developments in welding equipment and in the development of weldable structural materials. The introduction of higher-strength materials, with a higher proportion of alloying additives, is almost always associated with their impaired weldability, and requires close attention be paid to the effects that the welding process can have on the welded joint area. This often involves limiting the amount of heat introduced into the parts to be joined during welding. A measure of the amount of heat used to make a weld is the heat input.

Gas metal-arc welding (GMAW) is one of the most widely used welding processes. This method involves all types of shielding gases. Depending on the shielding gases used, GMAW welding can be divided into the following welding methods [1]:

- Metal inert gas (MIG) method—welding in a shield of chemically inert gases, such as Ar, He or mixtures thereof;
- Metal active gas (MAG) method—welding in a shield of chemically active gases, such as CO_2_, H_2_, O_2_, N_2_, and NO, used separately or as additives to Ar or He.

The GMAW welding process involves melting the edges of the workpieces to be joined and the fusible electrode material with the heat of a glowing electric arc, in a gas shield, between the fusible electrode and the material to be welded. The molten metal of the electrode wire passes into the welding pool. The metal of the welding pool, as the arc moves in the direction of welding, crystallizes to form a weld that joins the edges of the welded workpiece [1].

The fusible electrode is employed in the form of a solid wire with a diameter of 0.4–4 mm. It is fed continuously, at a constant speed of 1–50 m/min, through a feeding system. Current is supplied to the electrode wire through a current tip. GMAW torches can be water-cooled or air-cooled. The gas shielding of the welding arc ensures that the weld is formed under favorable thermal and metallurgical conditions.

When GMAW welding corrosion-resistant ferritic–austenitic stainless steels, the welding arc is energized with positive polarity (the upper electrode is the anode). If the polarity of the electrodes is reversed, the arc glows unstably and the metal spatter is large, with a small depth of remelting. Welding is carried out by short-circuit arc, spray arc or pulsed current [1,2].

The GMAW process allows for high welding speeds. Welding is carried out as semi-automatic mechanized, automatic, or in a robotic manner. Thanks to the high versatility of the process and the ease of adjustment, GMAW welding allows a wide variety of structures to be made of different metals and alloys under workshop and assembly conditions, in all positions. Increased efficiency can be achieved by increasing welding productivity, e.g., Twin Welding, Tandem Welding, TimeTwin Welding, SpeedPulse [1,3], as well as reducing the amount of heat input, e.g., Cold Metal Transfer, Surface Tension Transfer, Cold Arc and Controlled Bridge Transfer [4,5,6,7].

Heat input is a conceptual measure of the amount of energy delivered to a defined unit of weld length. During arc welding, the welding arc is responsible for transferring energy from the electrode to the weld piece. The function of the energy is to adequately melt the weld material in the weld zone, as well as the filler material (if used). Melting is accomplished by delivering the right amount of power (energy in a certain unit of time) and achieving the right level of energy density on the electrode. The heat input affects the cooling rate of the material, and thus the mechanical properties and structure of the weld and heat-affected zone [1,8,9].

Heat input cannot be measured directly—it is calculated based on the measured values of arc voltage, welding current and welding speed via the following Equation (1) [10]:

(1) $$Q=k\cdot \frac{U\cdot I}{v}\cdot 10^{-3}\;(kJ/mm).$$

where *Q*—heat input, kJ/mm; *k*—thermal efficiency coefficient of the welding process; *U*—arc voltage, V; *I*—welding current, A; *v*—welding speed, mm/s.

The values of thermal efficiency coefficient *k* for different welding methods are shown in Table 1.

During the welding process, there is a significant fluctuation of current parameters, which is a result of the high dynamics of the process due to the complexity of the phenomena occurring in the glowing welding arc. However, this variability is not considered when calculating the heat input (average values are usually considered) [11]. When pulsed current is used in the welding process, the average value of the current intensity is calculated using Equation (2) [12]:

(2) $$I_{av}=\frac{I_{i}\cdot t_{i}+I_{p}\cdot t_{p}}{t_{i}+t_{p}},\;A,$$

where *I_av_*—average current, A; *I_i_*—impulse current, A; *I_p_*—base current, A; *t_i_*—duration of the pulse current, ms; *t_p_*—duration of the base current, ms.

Heat input is considered a welding parameter that numerically describes the effect of the process on the properties of the welded material, and a parameter that allows for comparing with each other the effects of different welding processes (including pulse welding) on the weldability of the base material [11].

Currently, significant advances have been made involving the use, in welding equipment, of synergistic control systems, i.e., fast-acting elements that provide free shaping of the output characteristics, and the development of software that allows one to control the process by adjusting the shape of the current pulse waveform. This leads to a situation in which the classical approach to determining the heat input (based on its calculation using average values of current intensity and arc voltage read from conventional meters) can lead to its underestimation or overestimation, and consequently result in a lack of correlation between the value of heat input and the properties of the obtained joint [11,13,14]. Taking the above into account, ISO/TR 18491 [15] recommends using a data acquisition system capable of capturing sample values of arc voltage and welding current at a sampling rate that is at least 10 times the frequency of the signal waveform.

Ferritic–austenitic stainless steels are steels characterized by a two-phase structure (hence the name ‘duplex’). In a state of structural equilibrium, achieved by appropriate chemical composition and manufacturing processes, they have a fine-grained structure containing about 50% ferrite (*α*), while the rest is austenite (*γ*). Ferrite provides the steel with the required resistance to stress corrosion cracking and austenite with the appropriate plastic properties. These steels typically contain less than 0.03% carbon, 18.5–27% chromium, 1.5–3% molybdenum, 4–7% nickel, less than 2% copper and less than 0.2% nitrogen [16]. Duplex steels are an alternative to classical single-phase austenitic and ferritic steels, as they combine the good properties of both groups of these steels, maintaining high corrosion resistance and keeping the strength properties of ferritic stainless steels at a high level, with a relatively low coefficient of thermal expansion compared to austenitic stainless steels, along with a lower tendency towards grain growth. The duplex steel type already includes more than 40 grades, which are constantly being developed, and their corrosion resistance depends on the contents of alloying elements [2,17,18,19,20,21,22].

Ferritic–austenitic stainless steels are classified using the Pitting Resistance Equivalent (PRE) value as a criterion. This equivalent can take different formulas, the most widely used being Pitting Resistance Equivalent Number (PRE_N_), in the form of Equation (3) [16],

(3) $${PRE}_{N}=\%Cr+3.3\times \%Mo+16\times \%N.$$

In steels with added tungsten, the PRE_W_ equivalent of (4) is used [16],

(4) $$P{RE}_{W}=\%Cr+3.3\times (\%Mo+0.5\times \%W)+16\times \%N.$$

Based on the PRE_N_ value, ferritic–austenitic stainless steels can be divided into the following [23,24,25,26,27]:

- Lean duplex steels—PRE_N_ < 30, e.g., S32101 or S32304. They have higher manganese content, and lower chromium and nickel content than standard duplex steels, with little or no molybdenum. They are used in less aggressive environments and can be a cheaper alternative to austenitic corrosion-resistant steels, such as 304 L and 316 L;
- Duplex (standard) steels—PRE_N_ = 30–40, e.g., S32205 or S31803, containing 22% chromium and characterized by high corrosion resistance in aggressive environments, good mechanical properties and good weldability. This is the most widely used type of duplex steels, accounting for about 80% of the global use of these steels;
- Super duplex steels—PRE_N_ = 40–50, e.g., S32750 or S31260, containing 25% chromium and increased molybdenum and nitrogen content. Compared to standard duplex steels, they have higher corrosion resistance and enhanced mechanical properties, and can be used in warm seawater environments with high chlorine contents and in acidic chloride media (used for offshore structures, i.e., offshore exploration, mining, processing and transportation installations working on the high seas for the exploitation of mineral deposits located under the seabed);
- Hyper duplex steels—PREN > 50, e.g., S32707, high-alloy steels with increased chromium, molybdenum and nitrogen contents. These have very high resistance in chloride-containing environments with high mechanical properties, and are used for work in very aggressive corrosive conditions.

The higher the PRE_N_ value, the more resistant the steel is to corrosion. Steels with PRE_N_ values above 32 are considered resistant to seawater. The usefulness of the PRE_N_ index for estimating the resistance of welding materials is limited; these values should be considered only as comparative data, and the final selection of steel should be decided via tests in a specific corrosive medium and at a specific temperature [28].

Depending on the thermal cycle of welding, in the temperature range of 300–1000 °C, secondary phases varying in structure and chemical composition can be formed in duplex steels. The presence of these phases results in reduced impact strength and corrosion resistance. Due to the possibility of the occurrence of precipitation processes that depend on the welding thermal cycle, the welding parameters of ferritic–austenitic stainless steels must be strictly controlled, and welding processes should be carried out under conditions that ensure the minimization of the risk of the occurrence of intermetallic phases [29,30,31,32].

In scientific research and industrial practice, it is often necessary to determine the optimal parameter values for the various processes under study. If the mathematical form of the object and the relationships that exist in it are known, modeling and simulation are used, while where this is not possible, methods based on experience and on mathematical statistics are used, including statistical methods of experiment planning. In statistical methods of experiment planning, the assumption is made that a large part of cause-and-effect relationships can be explained by mathematical models in the form of functions that approximate test results. Statistical methods of experiment planning require the use of specially constructed experiment plans, which, by appropriate design, allow for the determination of mathematical models, characterized by a relatively high accuracy of prediction (forecasting), with a small number of experiments required. The statistical plans of experiments are also called response surface plans, since they make it possible to graphically present (using 2D and 3D graphs) the relationship between input variables and the output variable (system response). This makes it possible to analyze the observed changes in the value of the output quantity in a region limited to fixed ranges of the input variables [33,34].

Statistical plans of experiments are very rarely used to study and explain reaction mechanisms, but they can provide a starting point for optimizing process parameters. Their use makes it possible to converge only on a local optimum, but response surface plots make it possible to find the direction of the increase or decrease in the values tested, which increases the probability of quickly reaching the optimum in further experiments [35,36].

In planning experiments to study the relationship between independent variables and the dependent variable, regression analysis and mathematical statistics are used. Regression analysis makes it possible to obtain a regression function, which is a mathematical model built from the values of the input quantities and on the corresponding values of the output quantities. The importance of the mathematical model is that, using it, it is possible to solve process optimization tasks and predict the results of the process after changing the conditions of its implementation. The basis of the method of statistical planning of an experiment is the use of a simplified plan for the arrangement of experimental points in the factor space and the transition to a new coordinate system [34].

In recent years, there has also been growing interest in integrating artificial intelligence into welding processes. Studies [37,38] have shown that machine learning algorithms can be effectively used to optimize welding process parameters in real time.

There are few research results describing the relationship between the structures of the joints and their properties, as well as the input parameters of the welding process (current, arc voltage and welding speed, i.e., the amount of heat supplied to the joint) [39,40,41]. Previous research on the influence of welding parameters on the properties of welded joints focuses mainly on general relationships or selected aspects such as microstructure or corrosion resistance, while there is a lack of research describing the relationship between process parameters and the sizes of the cross-sectional areas of welds in duplex steel. Filling this gap will allow the development of predictive tools (that can predict process results) to support the design of welding processes and ensure high-quality welds in industrial applications. The literature lacks a systematic approach to determine the model relationships describing the effects of the input parameters of the welding process (current intensity, arc voltage and welding speed, that is, the amount of heat supplied to the joint) on the possibility of obtaining (with some probability) joints with the assumed cross-sectional area. Therefore, this study determines the relationship between the heat inputs and the sizes of the cross-sectional areas of joints made of ferritic–austenitic stainless steel by gas metal-arc welding. This relationship is particularly important in terms of the quality of the welded structure and the optimization of the welding process.

## 2. Materials and Methods

The purpose of the experimental research presented in this article was to determine the relationships between the values of the parameters included in heat input (welding current, arc voltage and welding speed) and their effects on the sizes of the cross-sectional areas of welds in joints made of ferritic–austenitic stainless steel using the GMAW method. An attempt was also made to determine to what extent it will be possible to predict the properties of fabricated welded joints using the functional relationship describing the effect of the value of heat input on the sizes of the cross-sectional areas of welds.

### 2.1. Research Plan

After the literature analysis and preliminary research, the factors present in the established research object were identified and divided into four groups:

1. Input quantities (observable, measurable, and controllable) that enable purposeful interaction with a research object;
2. Input quantities (observable, measurable but not controllable) that interfere with the test object;
3. Fixed input quantities (measurable and controllable) that define the conditions for the implementation of the process;
4. Output quantities (observable and measurable) which are the result of the values taken by the input quantities.

**Figure 1:**
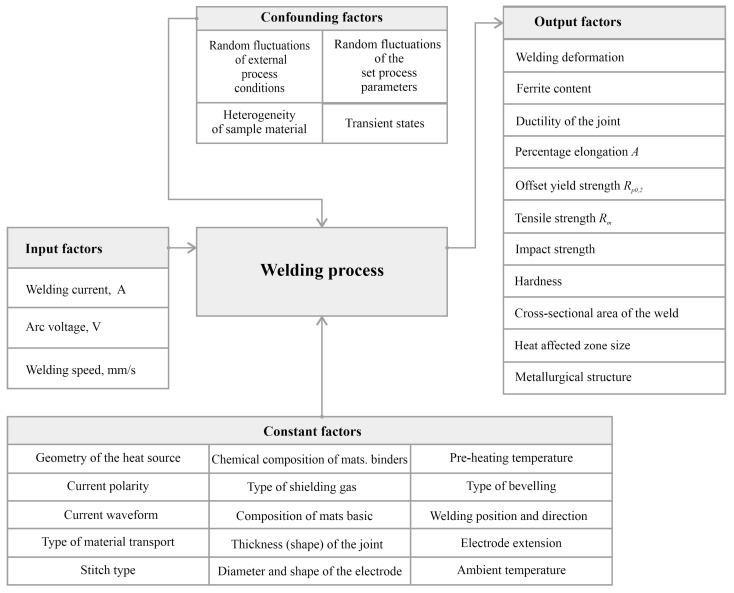
Diagram of the research object.

Figure 1 shows a schematic representation of the input, output, constants and confounding factors with respect to the test object.

The study was based on a five-level rotational experiment plan because of the increased precision of the model, the ability to model nonlinearity, the optimization of the scope of testing, its independence from the orientation of the axes of the coordinate system, and the uniform prediction accuracy. The choice of a five-level compositional rotational plan enables the accurate modeling of nonlinear relationships between the basic welding parameters, i.e., current, arc voltage and welding speed, and the weld cross-sectional area, minimizing the number of required experiments. The rotatability of the plan ensures uniform prediction (prediction) accuracy in all directions of the experimental space, which is crucial in studies with a wide variation of process parameters. This makes it possible not only to understand relationships, but also to optimize welding process parameters [33].

Since heat input is considered a welding parameter that numerically describes the effect of the process on the properties of the welded joint, to compare with each other the effects of different welding processes including pulse welding on the characteristics of the welded joint, the research plan was extended to perform additional tests on joints using the MAG (DC+) method and the MAG Pulse (DC+) method.

The ranges of variation of the input factors of the MAG (DC+) test joint welding process within the framework of the test plan (Table 2) were selected considering the recommendations of the filler metal manufacturer and available data from the literature.

Input parameters for the welding process of test joints using the MAG (DC+) method, according to the experimental plan, are shown in Table 3.

A summary of welding parameters for additional test joints using the MAG (DC+) method is presented in Table 4.

Table 5 shows a summary of the welding parameters of additional test joints using the MAG Pulse (DC+) method.

The selected current, voltage and welding speed ranges are well thought out and coincide with typical industrial conditions used in the welding of duplex steel. Their use in the study produces results that are not only valuable in a scientific context, but also practical in real industrial applications.

### 2.2. Materials

**Figure 2:**
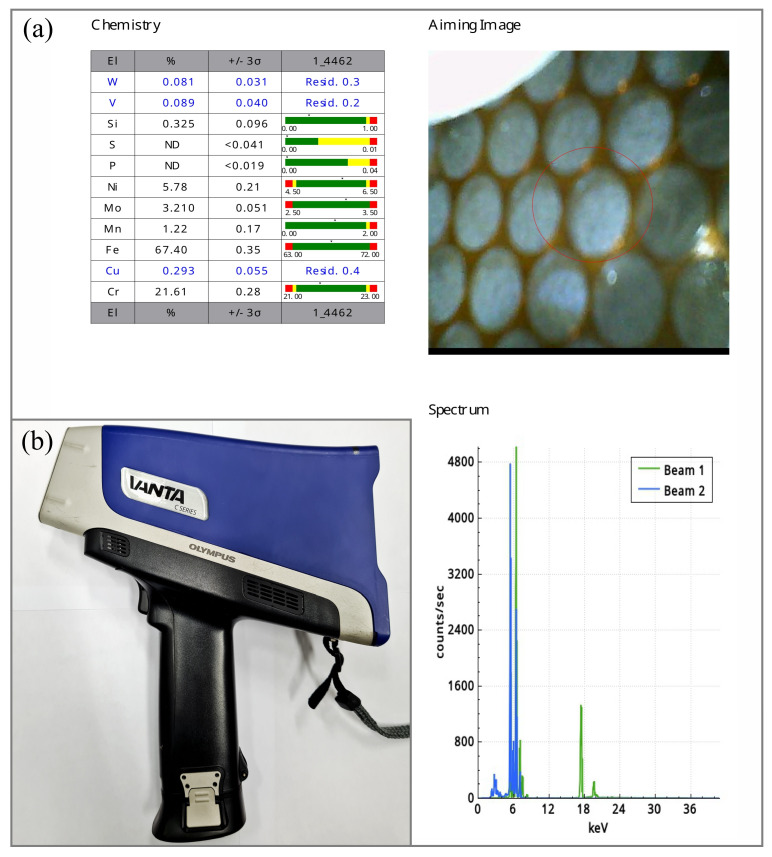
System for analyzing the chemical composition using the X-ray fluorescence method: (**a**) report on the analysis of the chemical composition of the steel used for testing; (**b**) VANTA XRF analyzer, VCR model, C series, Olympus.

A 3 × 1000 × 2000 mm sheet of duplex steel type 2205, produced by American Iron and Steel Institute (AISI) standard (X2CrNiMoN22-5-3, 1.4462 according to EN 10088-2 [42]), was used for the study. Manufacturer: Outokumpu Stainless AB (Helsinki, Finland), acceptance certificate 3.2 No. 6610/300425080, melt No. 571820-001. The sheet was delivered in a supersaturated condition at 1040 °C. A control chemical composition analysis was carried out using an Olympus XRF analyzer VANTA (Waltham, MA, USA), model VCR, C series (Figure 2).

X-ray fluorescence spectroscopy (XRF) is currently the most widely used analytical technique in non-destructive testing for the analysis of chemical composition. The XRF method is based on the fact that each element in the sample being analyzed, due to X-ray excitation, emits a characteristic spectrum that forms the basis for qualitative and quantitative analysis. The XRF analysis of chemical composition involves measuring secondary X-ray emission (fluorescence) from matter that has been excited by bombardment with high-energy X-rays or gamma radiation. The wavelength (energy) of the emitted fluorescence radiation characterizes the individual elements, while the measurement of the intensity of the radiation with a fixed wavelength (energy) makes it possible to determine the compactness of the element that emitted the radiation. Due to the speed of analysis and the possibility of making measurements without time-consuming sample preparation, XRF is widely used for quality control in metallurgy [43].

The essential technical data of the VANTA handheld XRF analyzer, model VCR (C series) from Olympus are included in Table 6.

The chemical composition of steel 2205 (1.4462) based on EN 10088-2 [42], acceptance certificate 3.2 and control analysis is shown in Table 7.

A 1.2 mm diameter AVESTA 2205 solid electrode wire (G 22 9 3 N L according to EN ISO 14343 [45], ER 2209 according to AWS A5.9) was used as the welding additive. The chemical composition of the welding wire is given in Table 8.

CRONIGON^®^ He20 (Pullach, Germany, M12-ArHeC-20/2 according to EN ISO 14175 [47]) with the following chemical composition was used as a shielding and forming gas: CO_2_ 2%; He 20%; Ar 78%.

For the tests, 66 specimens with dimensions of 3 × 150 × 350 mm were prepared in accordance with EN ISO 15614-1 [48] for 33 test joints. Due to the anisotropy of the mechanical properties (elongated grains and crystallographic structure formed during rolling), the samples were cut with the longer edge parallel to the rolling direction of the sheets. To reduce the amount of heat introduced into the base material, the cutting process of the specimens was performed using an abrasive water jet, and they were finished by milling.

### 2.3. Test Stand

**Figure 3:**
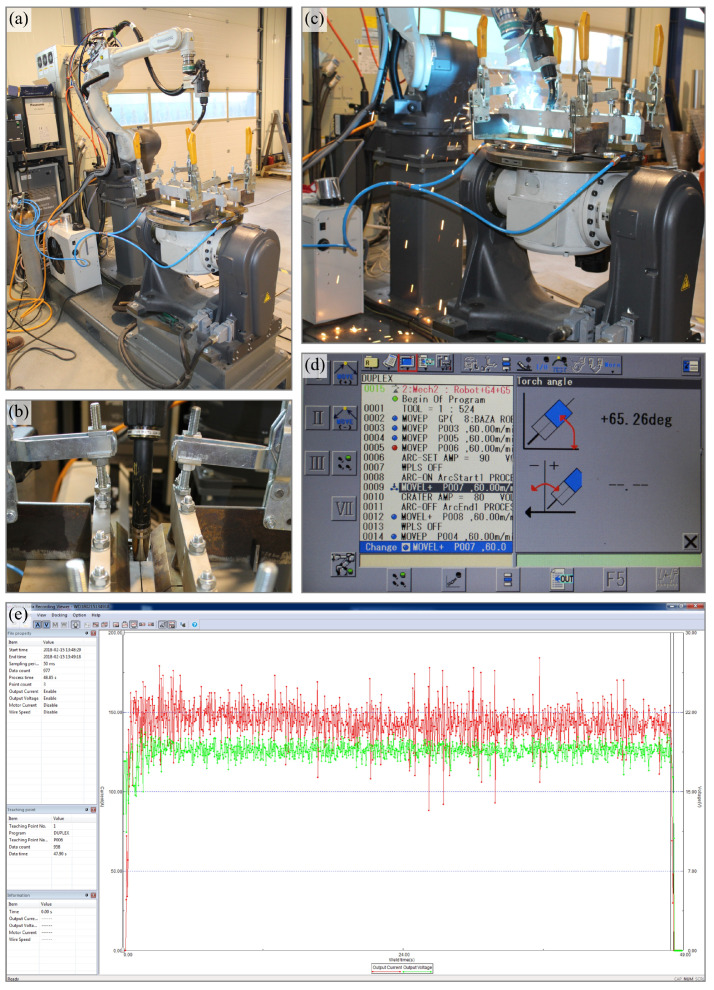
Test stand: (**a**) overall view; (**b**) specimen fixture and welding fixture orientation in a plane perpendicular to the welding direction; (**c**) test joint welding process; (**d**) view of welding fixture orientation settings screen; (**e**) view of the welding process parameters monitoring screen.

The robotic test stand was equipped with Panasonic’s TAWERS (The Arc Welding Robot System) welding system (Tokyo, Japan). This is an advanced technology platform that provides the necessary precision, stability and analytical capabilities needed to conduct experiments on the effects of welding parameters on weld quality. In a scientific context, the choice of such a system is apt and provides a significant advantage over more conventional methods. The innovation of the TAWERS system is related to the integration of the current source with the robot controller and the welding process monitoring system (Fusion 3 in 1). Such a solution results in a high speed of communication between the components of the test stand (72 Mbps). All controls are integrated on a 64-bit CPU PCB. The direct connection of controllers increases the speed of communication between controllers by more than 250 times compared to full digital communication systems. Information is exchanged every 10 μs. Such high-speed communication makes it possible to manage the main parameters of the welding process, i.e., arc voltage and current, and keep their average value practically constant, which translates into high quality welds [49]. Figure 3 shows the components of the test stand during the test joint welding process.

During the welding of the test joints, the energy parameters of the process, i.e., welding current and arc voltage, were measured and recorded based on signals transmitted from the measurement unit of the TAWERS welding system.

**Figure 4:**
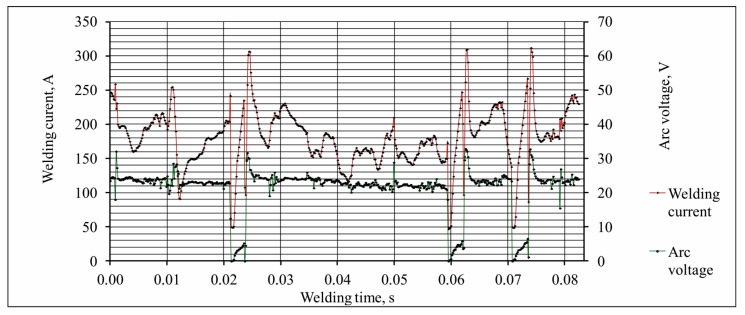
Example waveform showing instantaneous changes in welding current and arc voltage in the time domain—sampling frequency 6667 Hz (every 150 μs).

Based on the authors’ research and available literature data, the sampling frequency was determined, i.e., the number of measurements (samples) per unit time when converting a continuous analog signal into a discrete digital signal. For calculating the heat input of the process, data acquisition was performed at a frequency of 6667 Hz (Figure 4).

### 2.4. Station for Metallographic Macroscopic Examination of Welded Joints

**Figure 5:**
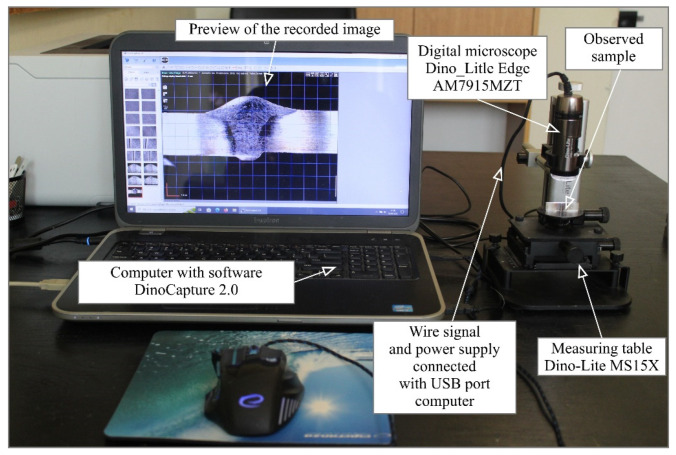
Station for metallographic macroscopic examination of welded joints equipped with a Dino Lite Edge AM7915MZT digital measuring microscope from AnMo Electronics Corporation.

To observe the macrostructure of the welded joints under study, a station equipped with a Dino-Lite Edge AM7915MZT digital measuring microscope from AnMo Electronics Corporation (New Taipei City, Taiwan) with dedicated DinoCapture 2.0 software was used (Figure 5).

The Dino-Lite Edge AM7915MZT digital measuring microscope can capture digital images of 5 Mpx (2592 × 1944 pixels) in visible light at a magnification of 10–230×. In addition, the device allows for recording video sequences (30 frames per second). The microscope is powered via a signal cable connected to the USB port of a PC. The light source of the AM7915MZT microscope is eight integrated LEDs (light-emitting diodes) with Flexible LED Control (FLC) illumination intensity control.

The DinoCapture 2.0 software allows microscope images to be recorded in EDOF (Extended Depth of Field) mode, which ensures that clear images are obtained even on very uneven surfaces. In addition, the software has a function for registering images in EDR (Extended Dynamic Range) mode, which allows us to expose brighter or darker areas of the registered image by superimposing images registered with different exposure times. The microscope is equipped with an AMR (Automatic Magnification Readout) system that automatically detects the magnification factor using software. A built-in adjustable polarizer allows us to remove unwanted reflection or glare on the object’s surface for better contrast. The specifications of the Dino-Lite Edge AM7915MZT digital measuring microscope are shown in Table 9.

## 3. Results and Discussion

**Figure 6:**
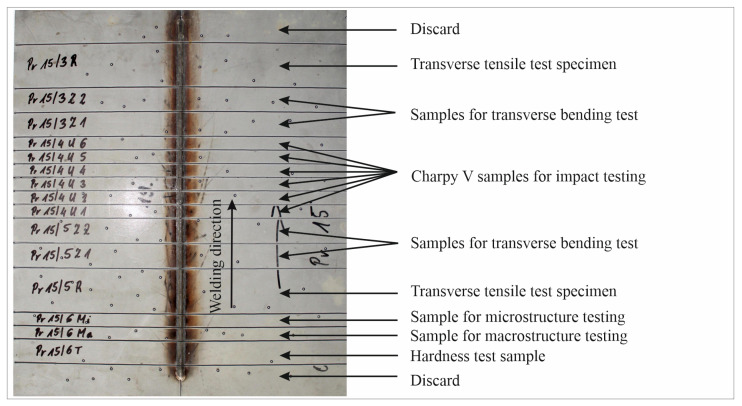
Arrangement of specimens for individual destructive tests according to EN ISO 15614-1 [48].

The basis for evaluating the macrostructure of a welded joint can only be the metallographic examination of a properly prepared sample. The locations of the specimens for the various destructive tests were chosen in accordance with welding practice and the EN ISO 15614-1 [48] standard, as illustrated in Figure 6.

To reduce the amount of heat introduced into the test joints, the cutting process of the specimens was carried out using a water-abrasive jet, followed by milling, grinding and polishing. The macrostructure of the joints was revealed by etching the surface of the test piece with Marble reagent (CuSO_4_ + HCl + H_2_O).

Macroscopic examinations were performed on the specimens for the entire cross-sections of the joints. They were conducted using a Dino-Lite Edge AM7915MZT digital measuring microscope and DinoCapture 2.0 software at 10× magnification.

Macroscopic examinations were performed to achieve the following [51]:

- Determine the type and shape of the joint;
- Determine the structure of the weld;
- Disclose the presence and identification of welding defects and non-conformities included in the EN ISO 5817: 2023 [52] standard;
- Determine the size and shape of the heat-affected zone;
- Disclose possible defects in the parent material.

**Figure 7:**
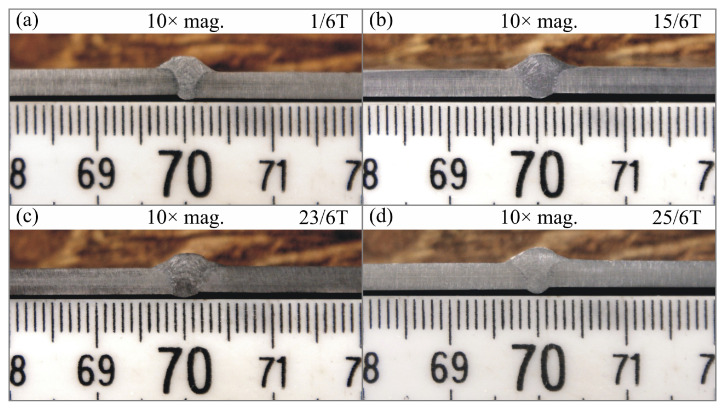
Cross-sectional view of selected test joints: (**a**) specimen 1/6 T; (**b**) specimen 1/15 T; (**c**) specimen 23/15 T; (**d**) specimen 25/15 T.

Macroscopic images of cross-sections of selected test joints (10× flood) are shown in Figure 7. The tests were performed in accordance with EN ISO 17639: 2013 [53].

In addition, the transverse fields of the welds (in five sections) were measured at 37.2× magnification.

Table 10 shows the results of macrostructure and cross-sectional area measurements of test joints made of 1.4462 steel, welded by MAG (DC+) with different values of heat input—the quality level of the joints was determined according to EN ISO 5817: 2023 [52].

Table 11 shows the results of macrostructure and cross-sectional area measurements of test joints of 1.4462 steel, welded using the MAG Pulse (DC+) method with different values of heat input—the quality level of the joints was determined according to EN ISO 5817: 2023 [52].

As a result of the non-destructive VT and RT tests, the quality level of the individual test joints, made of ferritic–austenitic stainless steel type 2205 according to AISI (1.4462 according to EN 10088-2 [42]), welded using the MAG (DC+) and MAG Pulse (DC+) methods, with different values of the basic process parameters, was determined according to EN ISO 5817: 2023 [52].

A total of 33 test joints were made, of which 15 met the quality level B criteria, while the remaining 18 joints did not meet quality level D. This high number of joints not meeting quality level D was due to the instability of the welding arc glow. The reason for the unstable arc glow was that the selection of welding parameters according to the experimental plan did not always take into account the physical conditions for the stability of the welding arc glow. As a result, too little or too much heat was introduced into some of the fabricated joints, causing unfavorable changes within the joint. By carrying out tests at all points in the experimental plan, it was necessary to determine the dependence of the expected value of the response function (regression function) on the experimental factors.

The predominant non-conformity of the test joints was related to a lack of meltdown (failure to melt the edges of the base material due to the lack of weld metal in the root), caused by insufficient heat input, which was insufficient to melt the edges of the welding groove, or by too much heat input causing the joint to burn through.

Analysis of the changes in the current and arc voltage during welding of the test joints recorded with a sampling frequency of 20 Hz showed a correlation between the changes in the welding arc state—characteristic of the transition from stable arc glow to unstable arc glow—and the resulting welding discrepancies. This allowed for locating areas in which the welding process was disrupted, resulting in welding discrepancies. The parameter whose changes indicate the possibility of welding discrepancies is the welding current.

In the area of the parent material, a two-phase ferritic–austenitic structure is visible, consisting of austenite grains in a ferritic matrix, which is characterized by a striated structure formed during the plastic processing (rolling) of the sheet. It is characterized by heterogeneity in grain size and shape, in which the austenite grains are elongated parallel to the rolling plane. The proportion of both phases is approximately 50%.

As the melting line is approached, i.e., in the heat-affected zone, the elongated austenite bands gradually disappear and the morphology of the interfacial boundary changes. In the heat-affected zone, large ferrite grains with a limited number of needle-like austenite precipitates are visible, which are mainly located at the boundaries of the ferrite grains and, to a lesser extent, inside the ferrite grains. The significant growth of ferrite grains in the heat-affected zone is a characteristic feature of welded joints in ferritic–austenitic stainless steels. As a result of the welding heat cycle, the heat-affected zone is heated to a temperature of approx. 1400 °C and acquires an almost entirely ferritic structure. During cooling, austenite is released. The process of forming a two-phase structure of ferrite (*α*) + austenite (*γ*) takes place at 1200–800 °C and, due to its diffusive character, its kinematics and effects depend on the cooling time of the steel in the above temperature range. Typical of the welding process, rapid cooling from a high temperature disrupts the transformation of ferrite to austenite, resulting in an increased proportion of ferrite. This is undesirable due to the risk of lowering the impact strength and corrosion resistance of the joints [16]. In the joints tested, the width of the heat-affected zone was small.

The kinematics of the phase transitions can be influenced by the welding technology. Under certain conditions of the welding process, the cooling rate, which determines the ferrite content, can be controlled by selecting the appropriate amount of heat input into the joint.

In joints of quality level B, the correct weld geometry was found. They are characterized by metallic continuity, a lack of cracks, inclusions and undercutting. In the remaining joints of quality levels C and D, the dominant welding defects were leaks in the root, incomplete penetration and incorrect weld edges.

**Figure 8:**
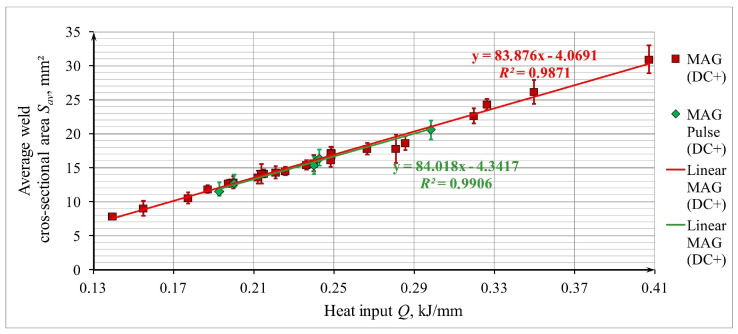
Plot of the dependence of the average weld cross-sectional area *S_av_* of 1.4462 steel test joints welded by MAG (DC+) and MAG Pulse (DC+) on heat input *Q*.

The correlation of the average cross-sectional area of welds *S_av_* and the amount of heat introduced into the joint (heat input *Q*) is shown in Figure 8—the drawing contains two trend lines (for different types of MAG welding).

The dependences of the average cross-sectional areas of the welds *S_av_* on the heat input *Q* in all the considered cross-sections were very similar, which allowed for the determination of changes in the tested samples. The course of the dependence of the average cross-sectional area of welds *S_av_* on the heat input *Q* is monotonically increasing and can be described by a linear function (an increase in the value of heat input causes a proportional increase in the value of the average cross-sectional area of welds).

- For the MAG method (DC+) (5),

(5) $$S_{av}=83.876\cdot Q-4.0691,$$

- For the MAG Pulse method (DC+) (6),

(6) $$S_{av}=84.018\cdot Q-4.3417,$$

where *S_av_*—average cross-sectional area of the weld, mm^2^; *Q*—heat input, kJ/mm.

The values of the determination coefficients (for Equation (5) *R*_2_ = 0.9871 and for Equation (6) *R*_2_ = 0.9906) prove a very good fit of the linear regression Equations (5) and (6) to the experimental data in the considered range of variability of the basic process parameters, and under the specific conditions set for its implementation.

Based on experimental data, using Statistica version 13 and Experiment Planner 1.0.1, a regression equation was determined describing the dependence of the average cross-sectional area of the weld *S_av_* on the given values of the input parameters of the welding process, adopting a dependence model in the form of an exponential function with an exponent in the form of an algebraic polynomial second degree, which has the following form:

(7) $$S_{av}=exp(-3.6282+0.04278\cdot I+0.42662\cdot U-5.2364\cdot v-0.0001142\cdot \;I^{2}-0.009164\cdot U^{2}+2.7576\cdot v^{2}),\;mm^{2},$$

where *S_av_*—average cross-sectional area of the weld, mm^2^; *U*—set arc voltage, V; *I*—set welding current, A; *v*—set welding speed, mm/s.

The calculated value of the coefficient of determination *R*^2^ = 0.9792 indicates a good fit of the exponential regression equation to the experimental data, as follows:

- Average value of all measurements, 16.929;
- Average value of the mathematical model results, 16.911;

The significance of the coefficient of determination *R*^2^ was checked with the *F* test (Wald test), finding the following:

- Value of the test function—*F* = 50.3567;
- Critical value of the statistic *F_cr_* for the significance level *α* = 0.05—*F_cr_* = 2.92.

Due to the fact that the inequality *F* > *F_cr_* occurs, there are no grounds to reject the hypothesis regarding the significance of the determination coefficient, i.e., the model fits to the experimental results for the assumed significance level *α* = 0.05.

Although the regression equation showed a good fit of the regression models to the experimental data, statistical validation was also carried out. The results of the measurements were checked for gross error using the Grubbs test (*t*-test). No results with gross error were found. The significance of the coefficient of determination *R*^2^ was checked using the *F*-Snedecor test, and it was found that there was no basis for rejecting the hypothesis of the significance of this coefficient. The adequacy of the mathematical model was also checked by estimating the variance of the measurement errors. The significance of the terms of the regression equation was also evaluated using the Student’s *t*-test.

The results of the cross-validation show that the average prediction error was 4%, and the analysis of the residuals confirms the absence of systematic deviations in the model. These results suggest that the regression models can be successfully used to predict the size of the weld cross-sectional area over the range of tested process parameters.

**Figure 9:**
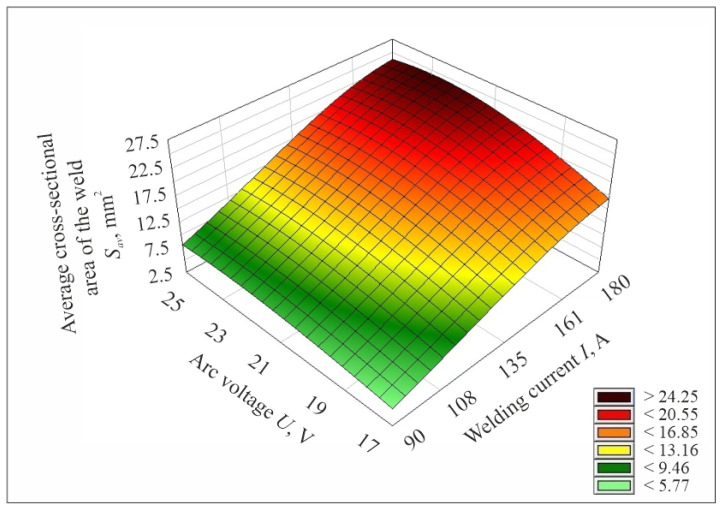
Plot of the mathematical model of the value of the average cross-sectional area of the weld *S_av_*, determined from experimental studies as a function of changes in welding current and arc voltage values for welding speed *v* = 10 mm/s.

**Figure 10:**
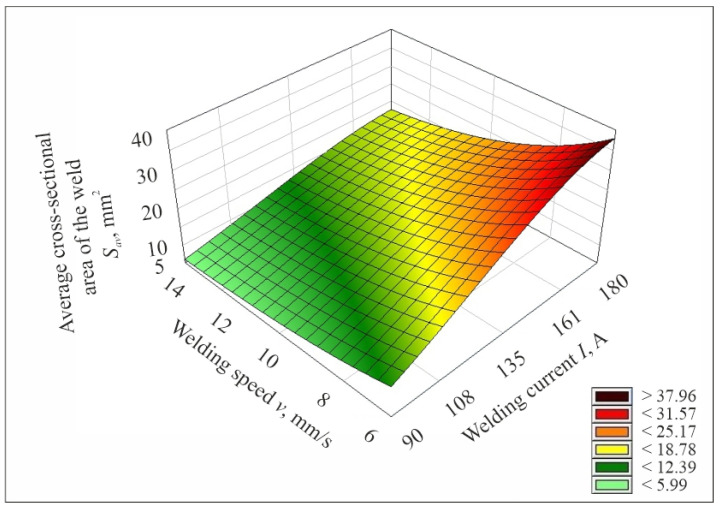
Plot of the mathematical model of the value of the average cross-sectional area of the weld *S_av_*, determined from experimental studies as a function of changes in welding current and welding speed for arc voltage *U* = 21 V.

**Figure 11:**
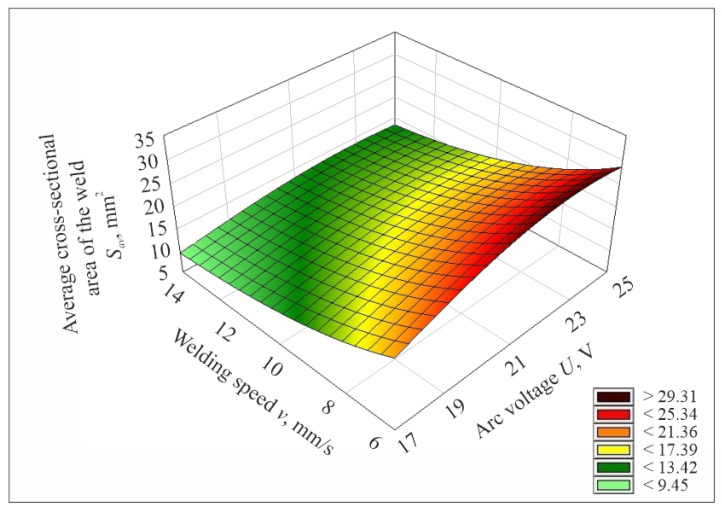
Plot of the mathematical model of the value of the average cross-sectional area of the weld *S_av_*, determined from experimental studies as a function of changes in arc voltage and welding speed for a welding current of *I* = 135 A.

After determining the mathematical model describing the studied process, its plots were prepared. Figure 9, Figure 10 and Figure 11 show the plotting of the mathematical model of the values of the average cross-sectional area of the weld *S_av_* determined by experimental tests, depending on the set values of the input parameters of the welding process, i.e., welding current, arc voltage and welding speed.

The experiments and evaluations of test joints of ferritic–austenitic stainless steel 1.4462 welded with different values of heat input using the MAG method (DC+) allowed for the determination of the permissible ranges of variation of the welding parameters, allowing for making joints meeting the requirements of quality level B according to EN ISO 5817. These ranges are as follows:

- Welding current—119.38–184.89 A;
- Arc voltage—16.92–23.16 V;
- Welding speed—6.67–12 mm/s;
- Heat input—0.1971–0.3262 kJ/mm.

## 4. Conclusions

The study included macrostructure evaluation and cross-sectional area measurements of MAG (DC+) and MAG Pulse (DC+) welded joints of ferritic–austenitic stainless steel (1.4462) with different heat input values. The results of the experimental studies allow us to formulate the following specific conclusions:

1. As a result of the experimental studies, the research hypotheses set were positively verified. The obtained test results confirm the existence of a correlation between heat input and the size of the average cross-sectional area of the weld, which is a representation of the volume of the melted metal, assuming a constant welding speed;
2. The test results and their analysis indicate that, based on the values of the input parameters of the welding process under study, the properties of the welded joints can be predicted;
3. The weld cross-sectional area *S_av_* (which is a representation of the volume of melted metal assuming a constant process speed) is an unambiguous measure of the heat input *Q* introduced into the joint in different varieties of MAG welding, i.e., MAG (DC+) and MAG Pulse (DC+);
4. The volume of the weld can be closely related to the amount of heat introduced into the joint during the welding process;
5. The weld cross-sectional area *S_av_* allows for a comparison of the actual thermal efficiency of different varieties of MAG welding;
6. The analysis of the developed mathematical models shows their suitability for explaining (and predicting) the sizes of the cross-sectional areas of welded joints depending on the values of the input parameters of the welding process;
7. Determining the regression function and making a three-dimensional plot of it (response surface) can provide a starting point for optimizing the parameters of the welding process;
8. The applicability of the relationships determined in this study is limited to the range of variability of the factors tested and the need to ensure the stability of arc glow and the production of joints that meet certain acceptance criteria, including the quality levels of welded joints and their strength properties.

The developed regression models can find practical applications in industry. They make it possible to predict the size of the weld cross-sectional area depending on process parameters, which supports the real-time optimization of welding parameters. In real-world applications, they can be used to improve weld quality by minimizing welding discrepancies, such as incomplete meltdown or joint burn-through, and to increase process efficiency. In addition, these models can support automated quality control systems in production lines, where it is crucial to ensure the repeatability and reliability of welded joints under industrial conditions. Their use is particularly important in sectors requiring high precision and durability, such as the offshore, chemical and energy industries. When combined with welding systems such as TAWERS, the models can be integrated as a tool for the ongoing monitoring and adjustment of process parameters, increasing the efficiency of production lines.

The experimental investigations carried out within the framework of this work and their analysis do not cover the entire range of issues related to the possibility of predicting the properties of welded joints based on the actual values of heat input and the relationships between the parameters included in its composition. It is recommended to continue research work in this area. Among the directions of further research, the following issues should be mentioned:

1. We should carry out studies on the effects of changes in the values of heat input and the parameters included in it, i.e., current intensity, arc voltage and welding speed, on selected properties of welded joints of different thicknesses, different joint types and different ferritic–austenitic stainless steels. This would allow the extrapolation of the experimental results. Experimental data with the actual value of heat input introduced into joints of a certain type, with a known thickness of the elements to be joined and made in known welding positions, and the actual values of the parameters included in it, for materials of known chemical composition, defined by ferrite number, could make it possible to determine ranges for the recommended values of process parameters;
2. Since the austenite content of a welded joint at ambient temperature with a given chemical composition of duplex steel depends almost exclusively on the cooling conditions (in gas-shielded welding it is also affected by the presence of nitrogen), it is advisable to carry out studies on the influence of the heat input and the values of the parameters involved on the cooling rate. By selecting an appropriate value for the heat input, the cooling rate can be influenced by the temperature range of the ferritic phase, and the mechanical properties of welded joints can be controlled.

## Figures and Tables

**Table 1:** Thermal efficiency factor, *k*, of welding process [10].

Process No.	Process	Factor *k*
121	Submerged arc welding with wire electrode	1.0
111	Metal-arc welding with covered electrode	0.8
131	MIG welding	0.8
135	MAG welding	0.8
114	Flux-cored wire metal-arc welding without gas shield	0.8
136	Flux-cored wire metal-arc welding with active gas shield	0.8
137	Flux-cored wire metal-arc welding with inert gas shield	0.8
138	Metal-cored wire metal-arc welding with active gas shield	0.8
139	Metal-cored wire metal-arc welding with inert gas shield	0.8
141	TIG welding	0.6
15	Plasma arc welding	0.6

**Table 2:** Input factors and ranges of their variation used for the study of the effect of changes in the value of heat input on selected properties of ferritic–austenitic stainless steel joints welded by the MAG (DC+) method.

Input Factors	Parameters of Input Factors
Weld symbol/welding position	BW (butt weld)/PA (downhand)
Welding current, A	90–180
Arc voltage, V	17–25
Welding speed, m/min	0.4–0.8
Shielding and forming gas flow rate, dm^3^/min	12/6

**Table 3:** Welding parameters of MAG (DC+) test joints according to the experimental test plan.

Experiment Number	Input Factors		
	Welding Current, A	Arc Voltage, V	Welding Speed, m/min
1	108	18.6	0.48
2	162	18.6	0.48
3	108	23.4	0.48
4	162	23.4	0.48
5	108	18.6	0.72
6	162	18.6	0.72
7	108	23.4	0.72
8	162	23.4	0.72
9	180	21.0	0.60
10	90	21.0	0.60
11	135	25.0	0.60
12	135	17.0	0.60
13	135	21.0	0.80
14	135	21.0	0.40
15	135	21.0	0.60
15.1	135	21.0	0.60
15.2	135	21.0	0.60
15.3	135	21.0	0.60
15.4	135	21.0	0.60
15.5	135	21.0	0.60

**Table 4:** Welding parameters for additional test joints using the MAG method (DC+).

Experiment Number	Input Factors		
	Welding Current, A	Arc Voltage, V	Welding Speed, m/min
16	130	16.0	0.50
17	140	16.0	0.50
18	108	18.6	0.60
19	120	18.6	0.60
21	120	20.1	0.60
23	140	21.6	0.60
25	110	19.6	0.48
29	90	20.4	0.35

**Table 5:** Welding parameters for additional test joints using the MAG Pulse (DC+) method.

Experiment Number	Input Factors			
	Welding Current, A	Time, ms	Arc Voltage, V	Welding Speed, m/min
20	*I_i_* = 126; *I_p_* = 114	*t_i_* = 15; *t_p_* = 15	20.1	0.60
22	*I_i_* = 148; *I_p_* = 132	*t_i_* = 15; *t_p_* = 15	21.6	0.60
24	*I_i_* = 120; *I_p_* = 100	*t_i_* = 15; *t_p_* = 15	19.6	0.48
26	*I_i_* = 190; *I_p_* = 170	*t_i_* = 15; *t_p_* = 15	23.1	0.70
28	*I_i_* = 93; *I_p_* = 79	*t_i_* = 15; *t_p_* = 15	20.4	0.35

**Table 6:** Technical specifications of the VANTA handheld XRF analyzer, model VCR, C series from Olympus [44].

Parameter	Specifications
Dimensions (W × H × D)	83 × 289 × 242 mm
Mass	1.70 kg with battery, 1.48 kg without battery
Excitation source	4 Watt X-ray tube; voltage, 8–50 kV
Primary beam filtration	8-position filter with automatic selection for each beam
Sensor	Silicon, semiconductor
Power supply	14.4 V lithium-ion rechargeable battery or 18 V power transformer 100–240 VAC, 50–60 Hz, 70 W max
Display	800 × 480 (WVGA) LCD with capacitive touch screen
Operating environment	Operating temperature range −10 °C to 50 °C; humidity, 10% to 90% relative humidity, non-condensing
Drop test	Military Standard 810-G 4-foot drop test (1.3 m drop test)
IP degree of protection	IP55: protection against dust and water jets from all directions
Pressure correction	Built-in barometer for automatic correction of air density
Operating system	Linux^®^; cloud-compatible
USB	2 USB 2.0 type A main ports; 1 USB 2.0 type mini-B port
Wireless LAN	Support 802.11 b/g/n (2.4 GHz), optional USB cable
Built-in camera	Full VGA resolution CMOS camera with autofocus lens
Data storage	Micro SD^TM^ slot with removable 1 GB SD card

**Table 7:** Chemical composition of duplex steel 2205 (1.4462).

	C, Mass%	Si, Mass%	Mn, Mass%	P, Mass%	S, Mass%	Cr, Mass%	Ni, Mass%	Mo, Mass%	Nb, Mass%	Cu, Mass%	Co, Mass%	N, Mass%
Requirements according to EN 10088-2	max. 0.03	max. 1.00	max. 2.00	max. 0.035	max. 0.015	21.0–23.0	4.50–6.50	2.50–3.50	–	–	–	0.10–0.22
According to acceptance certificate 3.2	0.02	0.44	1.34	0.029	0.001	22.22	5.69	3.13	0.007	0.28	0.160	0.167
According to control analysis	–	0.323	1.21	0.019	0.001	21.61	5.90	3.216	–	0.286	0.21	–

**Table 8:** Chemical composition of AVESTA 2205 solid electrode wire [46].

C, Mass%	Si, Mass%	Mn, Mass%	Cr, Mass%	Ni, Mass%	Mo, Mass%	N, Mass%	PRE_N_
≤0.015	0.40	1.70	22.50	8.80	3.20	0.15	≥35

**Table 9:** Technical parameters of the Dino-Lite Edge AM7915MZT digital measuring microscope from AnMo Electronics Corporation [50].

**Lighting**	Light source	LED
	Light color	White
	Number of diodes	8
	Switchable LEDs	Yes
**Lens**	Polarizer	Yes (linear)
	Magnification	10–230×
	Observation field	1.8 × 1.3 mm
	Lens type	Glass with an anti-reflective layer
**Matrix**	Matrix type	CMOS
	Resolution	5 Mpx (2592 × 1944 pixels)
	Frames per second	30
**Compatibility**	Interface	USB 2.0
	Operating system	Windows: XP, Vista, 7, 8, 10
	Software included	DinoCapture 2.0
	Supported image file formats	BMP; GIF; PNG; JPG; TIF; RAS; PNM; TGA; PCX; MNG; WBMP; JP2; JPC; PGX
	Supported video file formats	WMV; FLV; SWF
**Housing**	Housing material	Aluminum
	Zoom lock	Yes
	Dimensions	10.5 × 3.2 cm (length × diameter)
	Mass	137 g

**Table 10:** Results of macrostructure and cross-sectional area measurements of test joints of 1.4462 steel welded by MAG method (DC+) with different values of heat input.

Sample Number	Heat Input *Q*, kJ/mm	Joint Quality Level According to EN ISO 5817: 2023 [52]	Cross-Sectional Areas of the Weld *S*, mm^2^	Standard Deviation of the Average Weld Cross-Sectional Area, mm^2^	Macrophotographs of Joints (37.2× mag.)
1	*Q* = 0.2208	B	*S_1_* = 15.07 *S_2_* = 12.78 *S_3_* = 14.36 *S_4_* = 14.54 *S_5_* = 14.96 *S_av_* = 14.34	0.92	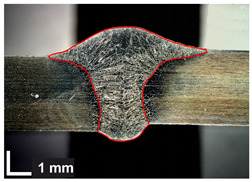
2	*Q* = 0.3496	C	*S_1_* = 24.89 *S_2_* = 29.10 *S_3_* = 26.77 *S_4_* = 25.01 *S_5_* = 25.32 *S_av_* = 26.22	1.78	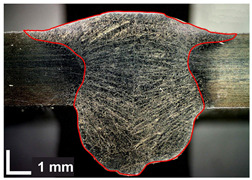
3	*Q* = 0.2398	B	*S_1_* = 16.57 *S_2_* = 13.72 *S_3_* = 15.56 *S_4_* = 16.44 *S_5_* = 16.24 *S_av_* = 15.71	1.18	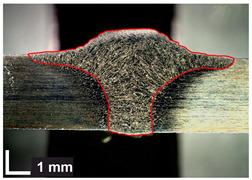
4	*Q* = 0.4070	D	*S_1_* = 29.43 *S_2_* = 32.54 *S_3_* = 28.97 *S_4_* = 30.23 *S_5_* = 33.76 *S_av_* = 30.99	2.07	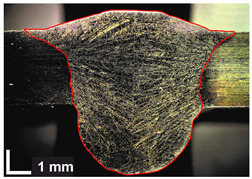
5	*Q* = 0.1393	D	*S_1_* = 7.81 *S_2_* = 8.24 *S_3_* = 7.41 *S_4_* = 7.65 *S_5_* = 8.36 *S_av_* = 7.89	0.40	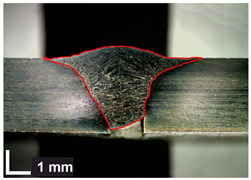
6	*Q* = 0.2149	B	*S_1_* = 14.97 *S_2_* = 14.34 *S_3_* = 13.98 *S_4_* = 13.43 *S_5_* = 14.27 *S_av_* = 14.20	0.56	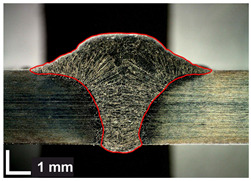
7	*Q* = 0.1548	D	*S_1_* = 9.78 *S_2_* = 7.28 *S_3_* = 8.87 *S_4_* = 9.57 *S_5_* = 9.89 *S_av_* = 9.08	1.08	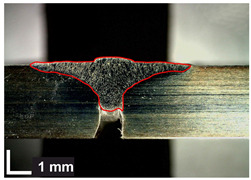
8	*Q* = 0.2854	B	*S_1_* = 20.22 *S_2_* = 17.56 *S_3_* = 19.14 *S_4_* = 18.22 *S_5_* = 18.32 *S_av_* = 18.69	1.02	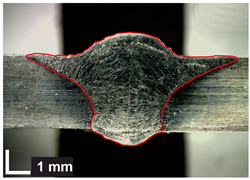
9	*Q* = 0.3196	B	*S_1_* = 23.76 *S_2_* = 23.84 *S_3_* = 22.57 *S_4_* = 21.87 *S_5_* = 21.34 *S_av_* = 22.68	1.12	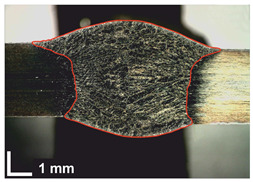
10	*Q* = 0.1395	D	*S_1_* = 7.60 *S_2_* = 7.96 *S_3_* = 7.86 *S_4_* = 7.87 *S_5_* = 8.23 *S_av_* = 7.90	0.23	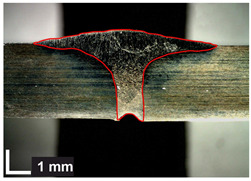
11	*Q* = 0.2807	B	*S_1_* = 16.93 *S_2_* = 14.75 *S_3_* = 19.78 *S_4_* = 18.23 *S_5_* = 19.56 *S_av_* = 17.85	2.08	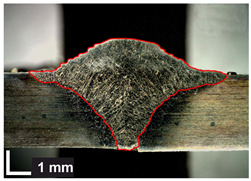
12	*Q* = 0.1869	D	*S_1_* = 12.81 *S_2_* = 11.86 *S_3_* = 12.02 *S_4_* = 11.36 *S_5_* = 11.42 *S_av_* = 11.89	0.58	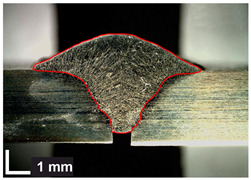
13	*Q* = 0.2256	B	*S_1_* = 14.92 *S_2_* = 14.75 *S_3_* = 13.61 *S_4_* = 15.22 *S_5_* = 14.32 *S_av_* = 14.56	0.62	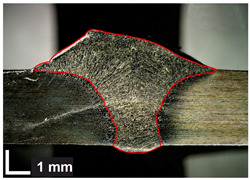
14	*Q* = 0.3262	B	*S_1_* = 25.54 *S_2_* = 24.96 *S_3_* = 23.56 *S_4_* = 23.98 *S_5_* = 23.81 *S_av_* = 24.37	0.84	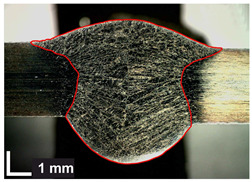
15	*Q* = 0.2485	B	*S_1_* = 18.12 *S_2_* = 16.27 *S_3_* = 16.42 *S_4_* = 17.04 *S_5_* = 16.51 *S_av_* = 16.87	0.76	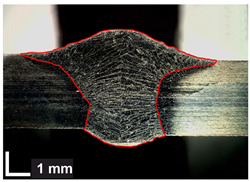
16	*Q* = 0.1997	D	*S_1_* = 14.37 *S_2_* = 13.07 *S_3_* = 12.13 *S_4_* = 12.42 *S_5_* = 12.54 *S_av_* = 12.91	0.89	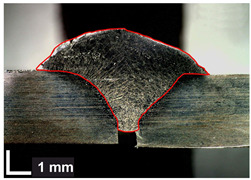
17	*Q* = 0.2137	D	*S_1_* = 16.18 *S_2_* = 15.13 *S_3_* = 13.28 *S_4_* = 13.11 *S_5_* = 13.06 *S_av_* = 14.15	1.42	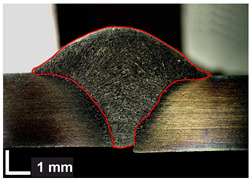
18	*Q* = 0.1771	D	*S_1_* = 11.93 *S_2_* = 9.97 *S_3_* = 10.36 *S_4_* = 10.54 *S_5_* = 10.12 *S_av_* = 10.58	0.78	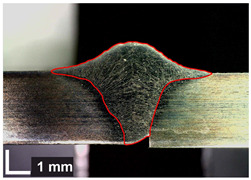
19	*Q* = 0.1971	B	*S_1_* = 12.37 *S_2_* = 12.81 *S_3_* = 13.22 *S_4_* = 12.01 *S_5_* = 13.34 *S_av_* = 12.75	0.56	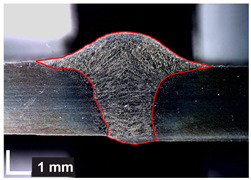
21	*Q* = 0.2119	B	*S_1_* = 14.38 *S_2_* = 12.14 *S_3_* = 13.34 *S_4_* = 13.89 *S_5_* = 14.44 *S_av_* = 13.64	0.95	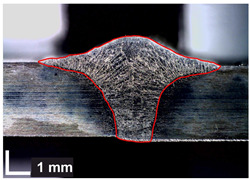
23	*Q* = 0.2664	B	*S_1_* = 19.26 *S_2_* = 17.43 *S_3_* = 18.11 *S_4_* = 17.36 *S_5_* = 17.01 *S_av_* = 17.83	0.89	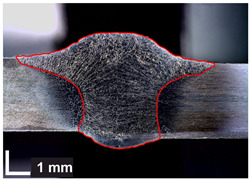
25	*Q* = 0.2361	B	*S_1_* = 16.48 *S_2_* = 14.82 *S_3_* = 15.84 *S_4_* = 15.22 *S_5_* = 14.96 *S_av_* = 15.46	0.69	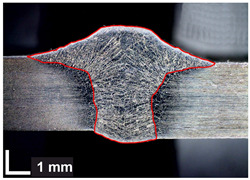
29	*Q* = 0.2483	D	*S_1_* = 15.52 *S_2_* = 14.67 *S_3_* = 16.84 *S_4_* = 16.86 *S_5_* = 16.94 *S_av_* = 16.17	1.02	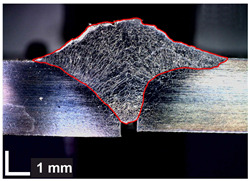

**Table 11:** Results of macrostructure and cross-sectional area measurements of test joints of 1.4462 steel welded by MAG Plus method (DC+) with different values of heat input.

Sample Number	Heat Input *Q*, kJ/mm	Joint Quality Level According to EN ISO 5817: 2023 [52]	Cross-Sectional Areas of the Weld *S*, mm^2^	Standard Deviation of the Average Weld Cross-Sectional Area, mm^2^	Macrophotographs of Joints (37.2× mag.)
20	*Q* = 0.1927	B	*S_1_* = 12.67 *S_2_* = 11.70 *S_3_* = 10.98 *S_4_* = 11.22 *S_5_* = 11.34 *S_av_* = 11.58	0.66	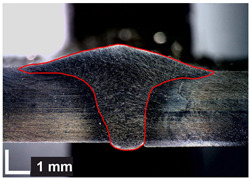
22	*Q* = 0.2426	D	*S_1_* = 16.52 *S_2_* = 18.15 *S_3_* = 16.22 *S_4_* = 15.89 *S_5_* = 15.31 *S_av_* = 16.42	1.07	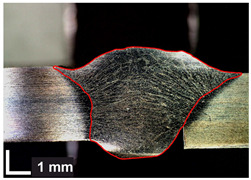
24	*Q* = 0.2001	B	*S_1_* = 13.08 *S_2_* = 13.21 *S_3_* = 12.41 *S_4_* = 11.86 *S_5_* = 13.35 *S_av_* = 12.78	0.63	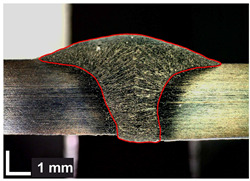
26	*Q* = 0.2982	B	*S_1_* = 22.05 *S_2_* = 18.32 *S_3_* = 21.68 *S_4_* = 20.98 *S_5_* = 20.43 *S_av_* = 20.69	1.47	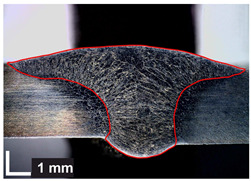
28	*Q* = 0.2399	B	*S_1_* = 14.54 *S_2_* = 13.91 *S_3_* = 16.72 *S_4_* = 16.68 *S_5_* = 15.21 *S_av_* = 15.41	1.26	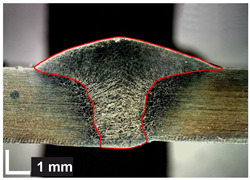

## Data Availability

The data presented in this study are available on request from the corresponding author because they require licensed software to process them.
